# Characteristics of Finnish and Swedish intensive care nursing narratives: a comparative analysis to support the development of clinical language technologies

**DOI:** 10.1186/2041-1480-2-S3-S1

**Published:** 2011-07-14

**Authors:** Helen Allvin, Elin Carlsson, Hercules Dalianis, Riitta Danielsson-Ojala, Vidas Daudaravičius, Martin Hassel, Dimitrios Kokkinakis, Heljä Lundgrén-Laine, Gunnar H Nilsson, Øystein Nytrø, Sanna Salanterä, Maria Skeppstedt, Hanna Suominen, Sumithra Velupillai

**Affiliations:** 1 Department of Computer and Systems Sciences (DSV), Stockholm University, Forum 100, SE-164 40 Kista, Sweden; 2 Department of Nursing Science, University of Turku and Hospital District of Southwest Finland, FI-20014 University of Turku, Turku, Finland; 3 Faculty of Informatics, Vytautas Magnus University, S. Daukanto g. 27 (301–309), LT-44249 Kaunas, Lithuania; 4 Department of Swedish, University of Gothenburg, Box 200, SE-405 30 Gothenburg, Sweden; 5 Department of Computer and Information Science, Norwegian University of Science and Technology, Sem Sælands vei 7-9, NO-7491 Trondheim, Norway; 6 NICTA, Canberra Research Laboratory and Australian National University, College of Engineering and Computer Science, Locked Bag 8001, ACT-2601, Canberra, Australia

## Abstract

**Background:**

Free text is helpful for entering information into electronic health records, but reusing it is a challenge. The need for language technology for processing Finnish and Swedish healthcare text is therefore evident; however, Finnish and Swedish are linguistically very dissimilar. In this paper we present a comparison of characteristics in Finnish and Swedish free-text nursing narratives from intensive care. This creates a framework for characterising and comparing clinical text and lays the groundwork for developing clinical language technologies.

**Methods:**

Our material included daily nursing narratives from one intensive care unit in Finland and one in Sweden. Inclusion criteria for patients were an inpatient period of least five days and an age of at least 16 years. We performed a comparative analysis as part of a collaborative effort between Finnish- and Swedish-speaking healthcare and language technology professionals that included both qualitative and quantitative aspects. The qualitative analysis addressed the content and structure of three average-sized health records from each country. In the quantitative analysis 514 Finnish and 379 Swedish health records were studied using various language technology tools.

**Results:**

Although the two languages are not closely related, nursing narratives in Finland and Sweden had many properties in common. Both made use of specialised jargon and their content was very similar. However, many of these characteristics were challenging regarding development of language technology to support producing and using clinical documentation.

**Conclusions:**

The way Finnish and Swedish intensive care nursing was documented, was not country or language dependent, but shared a common context, principles and structural features and even similar vocabulary elements. Technology solutions are therefore likely to be applicable to a wider range of natural languages, but they need linguistic tailoring.

**Availability:**

The Finnish and Swedish data can be found at: http://www.dsv.su.se/hexanord/data/.

## Background

The term *clinical text* stands for textual documents that are produced for clinical work which are often saved in clinical information systems [1,2]. The primary purpose of clinical text is to serve patient care as a summary or hand-over note, but clinical texts are also written to fulfil legal requirements and for purposes of reimbursement, management and research. The author can be a physician, nurse, therapist, specialist, or other clinician responsible for patient care. The text may have been entered into the system in real time, in retrospect, or as a summary made by the bedside or elsewhere, by the author or by a secretary who transcribes a dictation, by a speech recognition software, or by another system that generates or synthesises text. Clinical text applies to texts documenting the entire care process, and the actual content may differ substantially depending on the purpose – for example, describing the patient’s socio-medical history and current health problems as opposed to detailing care plans or even evaluating care outcomes. Synonyms or related terms include *case sheets*, *clinical data*, *clinical free text*, *clinical notes*, *clinical records*, *clinical reports*, *computer based patient records*, *digital patient records*, *discharge letters*, *discharge reports*, *discharge summaries*, *electronic health records*, *electronic patient records*, *health records*, *health reports*, *health text*, *medical records*, *medical reports*, *nursing discharge notes*, *nursing narratives*, *nursing notes*, *patient records*, and *patient’s chart*.

In several countries clinical documents are regulated by law and standardised via national or international models. In Finland, the legislation [3] stipulates that to ensure good care, clinical documents must cover all necessary information and the documents must adequately detail the patient’s conditions, care, and recovery. The text in the documents must be explicit, comprehensive, and include only generally well-known, accepted concepts and abbreviations. Swedish legislation has a similar approach [4].

In both Finland and Sweden, there are national models for *nursing narratives*, that is, clinical text written by nurses. Both models originate in the care process of gathering information from the patient, setting goals for care, implementing nursing interventions, and evaluating the outcomes of care. In Finland, a national standardised documentation model has been implemented that is based on the *Finnish care classification* (assessment, interventions, and outcomes of care) [5]. In Sweden, there is the *VIPS* (an acronym for the Swedish words for wellbeing, integrity, prevention, and security) *model*, which provides a structure for the documentation process with key words that reflect the nursing process [6].

In this paper we explore and compare the content and linguistic characteristics of nursing narratives from *intensive care units* (ICUs) with similar care systems but very different languages. Our analysis aims to support the development of clinical language technologies. The analysis is based on the *technology acceptance model *[*7*] with the hypothesis that perceived usefulness and ease-of-use are indicators of technology use. The analysis includes both a *qualitative* and a *quantitative approach*. The qualitative approach addresses document/technology usefulness by exploring the document content (i.e., what, when, why, from whom, to whom) and ease-of-use by analysing understandability and content accessibility. We extended this via the quantitative approach to problems in document accessibility and understandability. We performed the analysis with Finnish and Swedish data because of the differences between the two languages, but similarities between the two countries regarding healthcare and culture. We focused on ICUs – hospital units that provide 24/7 care for critically ill patients and focus on conditions that are life-threatening and require comprehensive care and constant monitoring – because of the similarity in ICU clinical decision-making processes between different nations and between different languages [8].

The criteria for intensive care admission, discharge, and triage are well defined in international guidelines [9,10] which standardises clinical decision-making processes in different ICUs. We used daily nursing narratives for the analysis because they cover the entire inpatient period.

## Methods

Our materials included daily nursing narratives from a Finnish and a Swedish ICU in university-affiliated hospitals [11]. Our inclusion criteria for patients were an ICU inpatient period of at least five days and an age of at least 16 years. Finnish (Swedish) health records were written between January 2005 and August 2006 (January 2006 and May 2008). Our research was approved by ethics committees in both countries (Ethics Committee of the Hospital District of South West Finland, 2/2009 §66 and the Ethics Committee in Stockholm, 2009/1742-31/5).

We analysed the materials by using *content analysis*, a widely used method for textual data which consists of systematic content coding with the aim of identifying themes and patterns in the data; the words and phrases mentioned most often are seen as those reflecting important concerns in communication [12-14]. We considered the daily nursing narratives as categorised data in which the content labels of the analysis correspond to the content headings written by the nurses. We compared these labels and contents with the aim of understanding their frequencies, contextual use, clarity, and relationships (e.g., parallel headings, synonymous concepts, negated concepts, subject-object roles, time order). Looking at the vocabulary and *n*-grams of different sizes generated from the whole data set, we explored the richness and expressive variation in the language and analysed the extent to which this posed a problem for the current context of the data set.

The analysis included both a qualitative and a quantitative approach. In the qualitative approach three average-sized health records from each data set (an average size of 2,389 and 5,169 words for Finland and Sweden, respectively) were used. The analysis was performed manually by three native Finnish speakers fluent in Swedish and two Swedish native speakers, four of whom are licensed healthcare professionals. The quantitative approach used 514 Finnish and 379 Swedish health records. For the Finnish data, we used the *FinTWOL morphological analyser with the FinCG disambiguator*[15], and for the Swedish data we used the *GTA*, *Granska Text Analyzer*[16]. When FinCG produced multiple alternatives (e.g., *haavan* [*wound’s*] → *haapa* [*aspen*] and *haava* [*wound*]) caused by highly inflective Finnish, we reduced the chances for sparse data by choosing only one alternative. The analysis was performed semi-automatically by a native Finnish speaker and a native Swedish speaker, both experts in clinical language technology development.

## Results

### Qualitative analysis

The documents contained notes from one professional to another in order to support information transfer and were similar in both countries and both languages (Table 1). They comprised key facts, reminders, and supplements to numeric data with a focus on changes in vital problems during the ongoing shift. Content themes included critical vital signs related to breathing, haemodynamics, temperature, diuresis, consciousness, pain, and medication administration. References to family members were common. In the Finnish data, the heading ***relatives*** was used in almost all daily narratives. The most common note was that next of kin had called during the shift. In the Swedish data, one of the obligatory headings was ***psychosocial background*** and nurses typically used this heading for notes concerning relatives. To illustrate differences in the data, the word *patient* or its abbreviation was used explicitly as a subject or object much more in the Swedish narratives than in the Finnish narratives.

**Table 1:** Special structural and contextual features of ICU nursing narratives.

Structural & contextual features	Finland (Finnish examples)	Sweden (Swedish examples)
**Headings**	**Headings are used in 2 of 3 health records. Headings are used as subjects and subjects are missing.** *Diuresis: occasionally profuse.* (*Diureesi: ajoittain runsasta.*) *Pupils move under eyelids but does not open eyes.* (*Pupillit liikkuvat luomien alla*, *mutta ei avaa silmiään.*)	**Headings are used in all narratives. The structure of headings seems to be obligatory. The headings are used as subjects.** *Circulation: Stable with inotropy.* (*Cirkulation: Stabil med inotropi.*) *Reacts only to pain stimulation during suctioning of intubation tube.* (*Reagerar enbart vid smärtstimuli vid sugning i tuben.*)

**Tense**	**Present and past participles are typical but *be*, *is* and *are* are not used. The most common tense is perfect.** *Consciousness remained unchanged.* (*Tajunta pysynyt ennallaan.*) *Blood pressure low.* (*Verenpaine matala.*)	**Present and past participles are typical but *be*, *is* and *are* are not used. The most common tense is perfect.** *Breathing: Ventilator parameters unchanged.* (*Andning: Ventilator parametrarna oförändrade.*)

**Structure of sentences**	**Complete sentences are rare.** *No spontaneous movements*, *rigidifies.* (*Ei spontaania liikettä*, *jäykistelee.*)	**Complete sentences are rare.** *Light sedation*, *looks up now and then.* (*Lätt sederad*, *tittar upp ibland.*).

**Misspelling**	**Misspellings exist but the meaning is clear.** *Henodynamics* (*Henodynamiikka*)	**Misspellings exist but the meaning is clear.** *The motther is informed.* (*Mammman är informered.*)

**Subjects (a patient)**	**The word *patient* as a subject is infrequently mentioned. If this word is mentioned it is not abbreviated.** *Oxygenates well or ventilates well.* (*Happeutuu hyvin tai ventiloituu hyvin.*)	**The word *patient* is used more often as a subject or object than in Finnish narratives. It is also replaced with abbreviations *Pat* or *Pt*. Use of *patient* was 40 % more common than *she*/*he*.** *Patient got a percutanous tracheostomy today.* (*Patienten har fått en perkutan trakeostomi idag.*) *Very worried about patient’s condition.* (*Mycket oroliga över patientens tillstånd.*) *Pt. wakes up when talked to and appears to be oriented.* (*Pt. vakner på tilltal och upplevs som adekvat.*)

**Signs and abbreviations**	**Signs and abbreviations are common. They originate from Finnish, Swedish, English, Latin, or professional jargon.** *The height of the drain rose from 10 --*>*20 mmHg.* (*Dreneerausrajaa nostettu 10 --*>*20 mmHg.*) *Got medicine --*>*good response.* (*Sai lääkettä --*>*hyvä vaste.*)	**Signs and abbreviations are common. They originate from Swedish, English, Latin, or professional jargon.** (*em*. [*eftermiddag*, *afternoon*]) *CVP* [*Central Venous Pressure*] *EN* [*Enteral Nutrition*] *TPN* [*Total Parenteral Nutrition*] *pO2* [*partial pressure of oxygen*] *pCO2* [*partial pressure of carbon dioxide*] *MAP* [*Mean Arterial Pressure*].

From the perspective of ease-of-use, analysts with ICU expertise considered the narratives to be clear and easy to understand. However, ICU-specific nonstandard abbreviations and acronyms were prevalent and some of them were unclear to analysts with less domain expertise. Consequently, narratives were difficult to understand for persons not working in specialised health care, especially for the patients and their relatives.

Using the documents was facilitated by content headings. Headings were used similarly in Finland and Sweden. Usually the content matched its heading; for example, ***Consciousness: ****Unchanged. Drain liquid brighter than yesterday*. In the Swedish data, content headings were obligatory and nurses selected them from a pre-defined list. They wrote their observations under the heading that was the closest match; for example, they wrote *body temperature* under the heading ***circulation*,** and *level of sedation* under the heading ***sleep***. In the Finnish data, reference resolution complicated content accessibility; nurses wrote headings freely and there were consequently numerous synonyms and closely related concepts; for example, ***haemodynamics*** – ***blood pressure*** – ***pulse*.** In addition, parts of the Finnish narratives were without headings. In that case, nurses either wrote their narratives in a story format with a clear plot or they started their notes with a word which can be considered as a heading (e.g. *Diuresis occasionally profuse*, *Therapeutic hypothermia still ongoing* or *Haemodynamic variation*)*.*

In addition to abbreviated words and problems with headings, reference resolution in the vocabulary as well as numerous linguistic and grammatical mistakes made using the documents difficult. For example, automated text analysis and reasoning seemed problematic with these data, with almost all sentences having no subject and approximately half of the sentences containing no verbs. The missing subject or object was usually the patient or clinician.

### Quantitative analysis

The most tangible problem in both data sets in terms of ease-of-use was reference resolution. The data sets were substantially rich in vocabulary, as demonstrated by the considerable amount of unique tokens as well as the fast convergence in common *n-*grams with increasing *n* (Table 2, Table 3, Table 4). Even though headings were established with respect to their content, their reference resolution in terms of naming conventions was prevalent (Table 5, Table 6). Words with complex spellings had innumerable variants (e.g. the word *Noradrenalin*, which had about 350 and 60 variations in the Finnish and Swedish data sets, respectively), while abbreviations/acronyms were nonstandard and ambiguous (e.g. *haemod* for *haemodynamics* and/or *haemodialysis*). Multiple terms were used for the same concept, and synonymous relations were often unclear (e.g. *breathing* – *oxidation* – *oxygenation – breath*). Problems related to missing subjects and objects were detectable due to the scarcity of pronouns when compared to the prevalence of verbs (Table 2). Further, detecting negated concepts is crucial for automated text analysis and reasoning; negations (e.g. *inte* and *ej* [*not*, Swe], and *ei* [*no*/*not*, Fin]) were among the most common types of words. However, temporal expressions (e.g. *time* and *evening*) were common in both data sets which suggests that tense analysis of verbs is unnecessary in developing language technologies.

**Table 2:** Quantitative comparison of ICU nursing narratives.

	Finland (Finnish)	Sweden (Swedish)
**Health records**	514 (496 unique patients)	379 (333 unique patients)
**Daily documents** (i.e., daily notes about a patient)	5,915 (17,103 shifts)	4,700
**Tokens**	1,227,909	1,959,271
**Types** (i.e., unique tokens) **before/after FinCG/GTA**	63,328 / 38,649	– / 41,883
**Tokens per patient / Tokens per daily document**		
**Minimum**	540 / 0	92 / 5
**Maximum**	14,118 / 915	36,830 / 9,389
**Average**	2,389 / 208	5,169 / 417
**Standard deviation**	1,635 / 87	5,271 / 239

**The number of bigrams**	368,166 (275,205 after FinCG)	469,455 (344,127 after GTA)
**The number of trigrams**	745,407 (356,307 after FinCG)	1,064,944 (905,539 after GTA)
**Proportion of pronouns after FinCG/GTA**	< 1%	2%
**Proportion of nouns after FinCG/GTA**	7%	27%
**Proportion of verbs after FinCG/GTA**	11%	11%

**Table 3:** The most common unigrams, bigrams and trigrams.

	Finland (Finnish)		Sweden (Swedish)	
**The most common unigrams after FinCG/GTA**	**unigram**	***n***	**unigram**	***n***
	*ja* [*and*]	28,628	*och* [*and*]	40,427
	*ei* [*no*]	20,557	*i* [*in*]	35,533
	*olla* [*be*]	15,452	*med*, [*with*]	32,568
	*saada* [*receive*]	10,995	*på* [*on*]	31,650
	*hapettua*[*oxygenate*]	10,665	*ha* [*have*]	22,633

**The most common bigrams after FinCG/GTA**	**bigram**	***n***	**bigram**	***n***
	*ei olla*	3,496	*circulation stabil*	3,775
	[*is not*]		[*circulation stabile*]	
	*hapettua hyvin*	2,517	*för att*	3,074
	[*oxygenate well*]		[*to*]	
	*yö aika*	1,475	*på morgon*	2,890
	[*night time*, misspelled]		[*in morning*]	
	*avata silmä*	1,299	*under natt*	2,792
	[*open eye*]		[*during night*]	
	*pitkä yö#vuoro*	1,144	*att suga*	2,648
	[*long night-shift*]		[*to suction* (*liquid*)]	

**The most common trigrams after FinCG/GTA**	**trigram**	***n***	**trigram**	***n***
	*hapettua ja tuulettua*	353	*i samband med*	1,958
	[*oxygenate and ventilate*]		[*in connection with*]	
	*ja tuulettua hyvin*	314	*slem att suga*	1,297
	[*and ventilate well*]		[*to suction secretions*]	
	*ei yhteyden#otto yö*	290	*munhåla och svalg*	1,189
	[*no contact night*]		[*oral cavity and throat*]	
	*ei olla tarvita*	279	*med god effect*	860
	[*have no need*]		[*with good effect*]	
	*yhteyden#otto yö aika*	264	*att suga i*	762
	[*contact night time*]		[*to suction in*]	

**Table 4:** The most common pronouns, nouns and verbs.

	Finland (Finnish)		Sweden (Swedish)	
**The most common pronouns after FinCG/GTA**	**pronoun**	***n***	**pronoun**	***n***
	*joka* [*which*]	3,166	*det* [*it*]	6,659
	*se* [*it*]	2,184	*han* [*him*]	4,707
	*tämä* [*this*]	1,354	*sig* [*them*(*selves*)]	4,656
	*mikä* [*that*]	452	*hon* [*her*]	3,908
	*ne* [*they*]	335	*detta* [*this*]	2,266

**The most common nouns after FinCG/GTA**	**noun**	***n***	**noun**	***n***
	*tajunta* [*consciousness*]	7,883	*andning* [*breathing*]	12,198
	*omainen* [*relative*]	6,301	*circulation* [*circulation*]	10,910
	*potilas* [*patient*]	6,242	*ml* [*ml*, abbr]	10,233
	*hengitys* [*breathing*]	6,242	*elimination* [*elimination*]	10,074
	*pulssi* [*pulse*]	5,722	*nutrition* [*nutrition*]	9,240

**The most common verbs after FinCG/GTA**	**verb**	***n***	**verb**	***n***
	*ei* [*no*]	20,557	*ha* [*have*]	22,633
	*olla* [*be*]	10,835	*vara* [*be*]	14,861
	*hapettua* [*oxygenate*]	9,269	*få* [*receive*]	11,975
	*saada* [*receive*]	3,879	*komma* [*come*]	4,569
	*soittaa* [*phone*]	3,622	*gå* [*walk*, *leave*]	4,460

**Table 5:** The most common topics.

Finland (Finnish)		Sweden (Swedish)	
**Topic**	***Approximate number of occurrences of each topic***	**Topic**	***Number of occurrences of each topic***

Haemodynamics	7,800	Respiratory	11,301
Consciousness	6,900	Circulation	10,630
Relatives	5,700	Elimination	10,041
Diuresis	5,400	Nutrition	8,258
Breathing	4,500	Communication	5,880
Oxygenation	3,600	Event time	5,681
Other	3,200	Pain	4,732
Excretion	590	Psychosocial	4,682
Haemodialysis	370	Sleep	4,438
Pulse	160	Skin	4,402
Skin	160	Activity	3,794

**Table 6:** The relations of the most common topics

Finland (Finnish)	Sweden (Swedish)
Haemodynamics Pulse	Circulation

Consciousness	Communication Pain Sleep Activity

Relatives	Psychosocial

Diuresis	Elimination

Breathing Oxygenation	Respiratory

Skin	Skin

To illustrate the need for domain-tailored technologies and resources, FinCG did not recognise 36 percent of the Finnish data (including punctuation). By tailoring the FinCG disambiguator with approximately 3,500 of the most common ICU terms, the method applicability improved substantially (see [17] and the references therein). The GTA handles unknown words differently than FinCG, but by comparing the ICU words with a general Swedish language corpus (PAROLE [18]), we estimated that 69 percent of the types were domain specific and thereby the need for domain-tailored methods was justified. Tailoring processes are likely to be similar for different languages and countries; words that were used for all patients and in all daily documents were very similar in both Finnish and Swedish data sets. These included the most common headings, temporal expressions, negations, and changes in observed patient state (e.g. *increase*, *continue*, *begin*). In these processes, which connect healthcare service providers, academic researchers, and commercial language and information systems providers, ensuring patient confidentiality is essential; the amount of protected health information was equal in the two data sets (1.5 person names per thousand words).

The most frequent tokens and types in a subset of the Finnish and Swedish data have been made publicly available [19].

## Discussion

In this paper we have presented a collaborative comparison of the content and linguistic characteristics in Finnish and Swedish nursing narratives taken from two national ICUs. There is a strong belief that capturing the clinical knowledge in such large-scale data sets could lead to improved safety and quality of care, promotion of clinical research and development of better language technology. However, although free text is helpful for entering information into clinical information systems, the complexity, variation and ambiguity of human languages make effective knowledge mining difficult.

Our results show that nonstandard headings, abbreviations, acronyms, and terminology complicate content accessibility. Similar results have been published for clinical text from US hospitals [20,21], from Finnish surgical, neurological, maternity and paediatric wards [22], from a medical-surgical ward in Thailand [23], and from Norwegian medical and cardiopulmonary units [24]. In addition, our results demonstrate that unclear and difficult-to-understand contents give rise to problems regarding document usefulness and ease-of-use. Previous studies have shown that both clinicians and patients have difficulties in interpreting clinical text, in particular abbreviations, medical terms and other professional jargon, and clinical reasoning [25,11]. Finally, the differences between general languages and domain jargon have been discussed in general (computational) linguistics studies, and it has been shown that the language of different specific domains or genres exhibits a high degree of linguistic variation [26,27].

The use of clinical text and knowledge mining can be supported by developing domain-tailored language technologies and resources that improve referential coherence in headings and vocabulary. International data standards, documentation models, and other standardisation resources include, for example, the *HL7 Health Level Seven International Standards *[*28*], *NANDA Nursing Diagnostic Terminology *[*29*], and *SNOMED CT Systematized Nomenclature of Medicine – Clinical Terms *[*30*]. As examples of technologies, we refer the reader to software for linguistic and grammatical proofing (e.g. *domain-tailored FinCG *[*17*,*31*]) and *Clinical Finnish Parser *[*32*], and methods for assigning headings automatically [17,33,34]. As examples of studies discussing the potential of language technologies to improve the clarity, understandability, and accessibility of clinical text for other languages, we refer the reader to studies [35] and [36] on English health sciences literature and clinical text, respectively.

However, the majority of content analyses and language technologies for clinical text consider only a monolingual level and do not compare other languages or countries with one another. Our paper explores and compares ICU nursing narratives in Finland and Sweden in both the Finnish and Swedish languages. Although the two languages are not closely related, nursing narratives in both languages have many characteristics in common, including similar content, structural features, and similar elements of vocabulary. We believe that this has implications for the design and development of common language technology solutions that support producing and using healthcare documentation in a better and more effective manner than is the case today. These common characteristics can also be interpreted as additional support for the similarities in clinical decision-making in ICUs (see [8]). To our knowledge, the 2007 study [37] is the only other paper comparing clinical text at a cross-lingual level (English, Japanese, Russian, Swedish) other than the conference version [38] of this paper.

Our study was limited to health records from only one ICU in each country, and these ICUs represented the highest level of intensive care. This may pose a problem regarding the representativeness of the data. The results of our study are not generalisable per se, but can be considered in Finnish and Swedish ICUs with similar care levels. Since there were many similarities between the Finnish and the Swedish ICUs, it is unlikely that different units with similar care levels within the countries have large differences. Finland and Sweden are closely related culturally but not linguistically. The cultural closeness might have affected the fact that the two different sets of text also seemed to be very similar in content and style.

The work presented in this paper represents merely a starting point and should be extended to other ICUs, clinics, languages, and countries. These extensions will enable us to analyse similarities and differences in clinical texts in a systematic way. We are also planning to carry out a more in-depth quantitative analysis by syntactic parsing of both sets of text. Moreover, we will study how to identify, normalise, and correct abbreviations and misspellings automatically by using various distance measures and concept-management techniques. We will also address the similarities and differences in clinical text written by various professional groups and at other hospital wards and healthcare units. Finally, we are eager to seek possibilities to incorporate laypeople’s information needs, and their interaction with healthcare providers, in our study.

## Conclusions

In our study the way Finnish and Swedish intensive care nursing was documented was not country or language dependent, but shared several common contexts, principles, structural features and even similar vocabulary elements. For example, both Finnish and Swedish data showed a lack of subjects and a substantial amount of non-standard abbreviations. We are therefore convinced that language technology solutions are likely to be applicable to a wider range of natural languages and to be very useful in the clinical setting. However, the technologies still need linguistic tailoring, and for wider applicability, multi-lingual analyses are needed. The framework we have introduced for analysing and comparing clinical text is practical and applicable for similar studies.

## Competing interests

The authors declare that they have no competing interests.

## Authors' contributions

All authors contributed to the study design and commented on the manuscript. HS coordinated the collaborative writing process and drafted the final manuscript. HD initiated the research work and did part of the background and discussion sections together with DK, EC, GN, HL-L, MS, ØN, SS, and VD. GN, HA, HL-L, RD-O, and SS carried out the qualitative analysis and HS, MH, and SV performed the quantitative analysis.
